# Syk and Hrs Regulate TLR3-Mediated Antiviral Response in Murine Astrocytes

**DOI:** 10.1155/2019/6927380

**Published:** 2019-04-04

**Authors:** Matylda B. Mielcarska, Magdalena Bossowska-Nowicka, Karolina P. Gregorczyk-Zboroch, Zbigniew Wyżewski, Lidia Szulc-Dąbrowska, Małgorzata Gieryńska, Felix N. Toka

**Affiliations:** ^1^Division of Immunology, Department of Preclinical Sciences, Faculty of Veterinary Medicine, Warsaw University of Life Sciences, Ciszewskiego 8, 02-786 Warsaw, Poland; ^2^Department of Biochemistry, Faculty of Agriculture and Biology, Warsaw University of Life Sciences, Nowoursynowska 159, 02-776 Warsaw, Poland; ^3^Center for Integrative Mammalian Research, Department of Biomedical Sciences, Ross University School of Veterinary Medicine, PO Box 334, Basseterre, Saint Kitts and Nevis

## Abstract

Toll-like receptors (TLRs) sense the presence of pathogen-associated molecular patterns. Nevertheless, the mechanisms modulating TLR-triggered innate immune responses are not yet fully understood. Complex regulatory systems exist to appropriately direct immune responses against foreign or self-nucleic acids, and a critical role of hepatocyte growth factor-regulated tyrosine kinase substrate (HRS), endosomal sorting complex required for transportation-0 (ESCRT-0) subunit, has recently been implicated in the endolysosomal transportation of TLR7 and TLR9. We investigated the involvement of Syk, Hrs, and STAM in the regulation of the TLR3 signaling pathway in a murine astrocyte cell line C8-D1A following cell stimulation with a viral dsRNA mimetic. Our data uncover a relationship between TLR3 and ESCRT-0, point out Syk as dsRNA-activated kinase, and suggest the role for Syk in mediating TLR3 signaling in murine astrocytes. We show molecular events that occur shortly after dsRNA stimulation of astrocytes and result in Syk Tyr-342 phosphorylation. Further, TLR3 undergoes proteolytic processing; the resulting TLR3 N-terminal form interacts with Hrs. The knockdown of Syk and Hrs enhances TLR3-mediated antiviral response in the form of IFN-*β*, IL-6, and CXCL8 secretion. Understanding the role of Syk and Hrs in TLR3 immune responses is of high importance since activation and precise execution of the TLR3 signaling pathway in the brain seem to be particularly significant in mounting an effective antiviral defense. Infection of the brain with herpes simplex type 1 virus may increase the secretion of amyloid-*β* by neurons and astrocytes and be a causal factor in degenerative diseases such as Alzheimer's disease. Errors in TLR3 signaling, especially related to the precise regulation of the receptor transportation and degradation, need careful observation as they may disclose foundations to identify novel or sustain known therapeutic targets.

## 1. Introduction

Astrocytes constitute 19-40% of brain glial cells and are the key component responsible for homeostasis and immune and oxidative stress defense in the CNS [1, 2]. By participating in the biogenesis and transport of a wide range of neuroactive substances, they affect neurons and other glial cells and thus regulate many physiological and pathophysiological processes [3]. In neuropathological conditions, immunologically silent astrocytes often undergo reactive reprogramming [4].

Recent reports reveal astrocytes as cells that play a substantial role in the pathogenesis of Alzheimer's disease (AD), the most frequent type of brain amyloidosis and the most common type of dementia in humans [5]. Amyloid-*β* (A*β*) plaques, AD's hallmark, activate cerebral glial cells and cause neuroinflammation resulting in neuronal cell death [6]. Such a process is decisive for the progression of AD. An increase in the number of reactive astrocytes that surround and may phagocytose A*β* plaques is observed in the neuronal vicinity [7]. However, following reactive reprogramming, astrocytes exhibit high concentrations of the amyloid precursor protein (APP) and *β*- and *γ*-secretase that enable formulation of the A*β* plaques [6]. Interestingly, during reactive gliosis, about 50% of the altered gene expression in astrocytes is significantly dependent on the initiating brain injury [8]. One of the factors leading to neuroinflammation, which may contribute to astrocyte reprogramming and enhanced astrocytic secretion of A*β*, is herpes simplex type 1 virus (HSV-1) infection in the brain. A*β* accumulates in HSV-1-infected cell cultures, while viral particles, as well as viral nucleic acid were found in the vicinity of amyloid plaques in the brains of mice and humans [9]. Toll-like receptor 3 (TLR3) plays an essential role in the innate immune control of cerebral HSV-1 infection. Therefore, it is likely that infected astrocytes detect the virus through TLR3, thus activating them and contributing to production of A*β*.

Substantial expression of TLR3 occurs in neurons and astrocytes, oligodendrocytes, and microglia, of which expression in astrocytes is the most abundant [10, 11]. An increase in the TLR3 expression in CNS-resident cells is usually associated with the development of neuroinflammation [12]. Stimulated glial cells and macrophages are responsible for the removal of microorganisms and injured cells. In addition to the production of various growth factors, chemokines and cytokines such as IL-1*β*, TNF-*α*, IL-6, and glial cells secrete oxides that may become neurotoxic during brain injuries or neurological diseases and exacerbate CNS dysfunction [13, 14].

Toll-like receptors (TLRs) are important contributors to activation of the innate immune response in the brain during infection, injury, or degeneration [15–18]. TLR3 is an evolutionarily conserved protein which recognizes double-stranded RNA (dsRNA) in endosomes. Double-stranded RNA may constitute a viral nucleic acid or an intermediate product formed during replication of viruses such as HSV-1 and HSV-2. Patients, especially children with deficiencies in TLR3 or single-gene errors in components of the TLR3 signaling pathway, are more susceptible to HSV-1 and HSV-2 infections, which may be the cause of a devastating human disease—herpes simplex encephalitis (HSE) [19–22]. Being one of the most common viral brain diseases in the world, HSE entails many deleterious outcomes. The accession of external factors into the central nervous system (CNS) and the CNS immune response are precisely controlled; however, due to the neuronal latency of HSV, inflammation in the brain may last for years and have a recurrent character [23]. The length of inflammatory response in CNS and disease progression is affected by the balance between pro- and anti-inflammatory signals in the neuronal environment [24]. Sustainability of the inflammatory process or deficiencies in distribution of suppressive mechanisms may lead to pathological repercussions and influence the outcome of disease.

Prior to launching the signaling cascade, nucleic acid-sensingTLRs such as TLR3, TLR7, and TLR9 enter the UNC93B1-dependent secretory pathway from the endoplasmic reticulum (ER) through the trans-Golgi network (TGN) to endosomes. However, receptors are subject to differential UNC93B1-related sorting mechanisms [25]. Furthermore, the endosomal sorting complex required for transport-0 (ESCRT-0), composed of hepatocyte growth factor-regulated tyrosine kinase substrate (Hrs) and signal transducing adaptor molecule (STAM), was recently implicated in post-Golgi trafficking by sorting ubiquitinated TLR7 and TLR9 to endosomes [26], and silencing of Hrs reduced signaling through TLR7 and TLR9 [27]. Chiang et al. [27] indicated that Hrs binds directly to particular TLRs and that the interaction of Hrs with TLR9 was much stronger than that with cell-surface-expressed TLR2. STAM, similar to Hrs, may demonstrate endosomal localization and display a potent sorting efficiency due to multiple ubiquitin-binding domains (UBDs). Moreover, it was demonstrated that STAM localizes prominently to early endocytic vesicles and decidedly regulates morphology of the Golgi apparatus [28], the site where TLRs are packaged en route to endosomes.

In addition to TLR trafficking aimed at ligand recognition, ESCRT-0-mediated sorting of receptors may direct them for recycling or degradation. Regardless of ligand stimulation, IL-2 receptor *β* and IL-4 receptor *α* were consistently internalized and delivered to late endosomes (LE) in an ESCRT-dependent manner by association with Hrs [29]. Following activation, receptors such as EGFR, PDGF, or TLR4 were endocytosed and targeted via the ESCRT pathway for lysosomal degradation [30–32]. The formation of the endosomal sorting machinery and its ability to target EGFR were regulated in this case by modulation of Hrs protein level, phosphorylation, and ubiquitination [33]. Furthermore, deficiencies and overexpression of ESCRT machinery components led to reduced EGFR degradation [34]. Because EGFR is responsible for TLR3 phosphorylation, posttranslational modifications, as well as alterations in the expression of ESCRT-0 subunits could affect TLR3 signaling.

**Figure 1:**
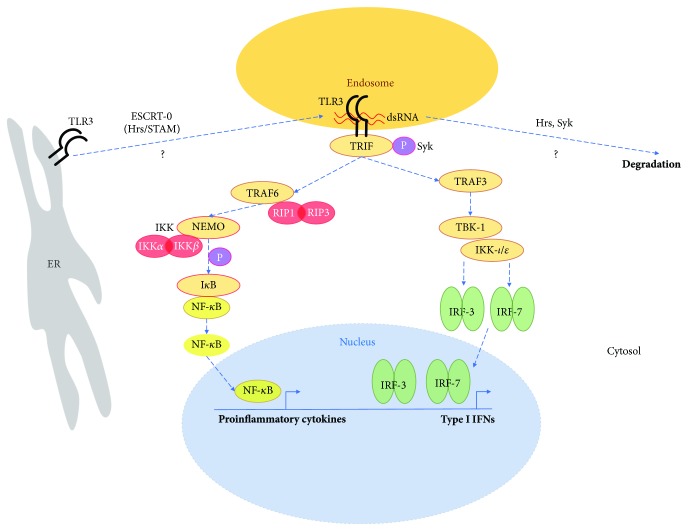
TLR3 signaling in astrocytes. Upon dsRNA recognition in the endosomal compartment, TLR3 undergoes dimerization and interacts with the TRIF adaptor molecule. TRIF activation is followed by TRAF6 and TRAF3 recruitment. TRAF6 conducts the signal *via* RIP-1 and RIP-3 kinases which facilitate NEMO, IKK-*α*, and IKK-*β* complex formation, followed by NF-*κ*B phosphorylation and translocation into the nucleus. TRAF3 engages TBK1 and IKK-i/*Ɛ* for IRF3 and IRF7 activation, followed by their dimerization and translocation into the nucleus. This leads to the induction of type I IFNs and proinflammatory cytokine gene expression. The dotted arrows highlight possible roles of ESCRT-0 in TLR3 transport from the ER to the endosome, as well as the role of Hrs and Syk in TLR3 degradation.

During HSV infection, release of viral dsRNA from the cells at the site of brain injury entails TLR3 activation. Upon phosphorylation by Bruton tyrosine kinase (Btk), c-terminal Src kinase (c-Src), and epidermal growth factor receptor (EGFR), the receptor triggers signaling in a pathway that enrolls transcription factors such as nuclear factor kappa B (NF-*κ*B), interferon regulatory factor 3 (IRF3), and interferon regulatory factor 7 (IRF7), responsible for the development of inflammatory response [35–37] (Figure 1). Compared to other endosomal TLRs, TLR3 engages a different adaptor protein (Toll/interleukin 1 receptor domain-containing adaptor protein inducing IFN-*β* (TRIF)) for initiation of IRF3 and NF-*κ*B signaling. Spleen tyrosine kinase (Syk) has been shown to phosphorylate tyrosine residues of TRIF, the TLR3 adaptor protein. Such a process leads to the TRIF proteasomal degradation and entails downregulation of the TLR signaling [38]. Furthermore, Syk significantly regulates Hrs phosphorylation and ubiquitination, as well as its membrane/cytosol localization [39]. Syk is known to function at the plasma membrane, but also in cytoplasmic and nuclear compartments of the cells [40], while Hrs may interact with Golgi proteins or reside in membranes of early endosomes and mediate delivery of protein cargo to multivesicular bodies (MVBs) for the subsequent degradation [41]. Consequently, Hrs serves as an important trafficking regulator and both Syk and Hrs may be critical controllers of protein entry into lysosomes for degradation. However, influence of these proteins on TLR3 signaling in CNS cells remains largely unexplored. The impact of Syk and ESCRT-0 on activation of the immune response may be receptor- and pathway-specific. It may be distinctively regulated in various cellular compartments and types of cells or tissues, as the ESCRT-0 target proteins may fulfill their function in the endosomes or on the cell surface.

Progression of the antiviral defense is guided by increased levels of type I interferons (IFNs) (IFN*α*, IFN*β*), cytokines (IL-1*β*, IL-6, and TNF*α*), chemokines (CXCL8, CCL5, and CXCL10), and other molecules, such as 2′5′OAS. The ground for the clinical signs of HSE is as follows: impaired but not abolished IFN*α*/*β* and IFN*γ* production in response to TLR3 stimulation [42]. Because HSE is manifested in CNS, attention should be paid to discovering and characterizing immunological engagement in HSV-1 and HSV-2 control, particularly related to TLR3 transportation, activation, and degradation in CNS-resident cells.

In this research, we investigated the involvement of Syk, Hrs, and STAM in the regulation of the TLR3 signaling pathway in the C8-D1A cell line. Our studies identify molecular events in murine astrocytes such as phosphorylation of Syk and Hrs and interaction of Syk and Hrs that occur shortly after TLR3 stimulation. We also show that the receptor undergoes ligand-induced proteolytic processing and that the N-terminal form of TLR3 exclusively interacts with Hrs. Finally, we demonstrate that silencing of Syk or Hrs in astrocytes significantly upregulates TLR3-directed signaling, indicating these proteins as targets for modulating TLR3 immune responses.

## 2. Materials and Methods

### 2.1. Cell Culture

Murine astrocytes from the C8-D1A cell line (ATCC® CRL-2541, Manassas, VA, USA) were used in all experiments. Cells were cultured in DMEM with high glucose and supplemented with 4.0 mM L-glutamine medium (Sigma-Aldrich, St. Louis, MO, USA), 10% heat-inactivated FBS (Sigma-Aldrich), and 1% solution of penicillin G, streptomycin, and amphotericin B (Sigma-Aldrich), in a humidified 5% CO_2_ incubator at 37°C. Astrocytes were subcultured according to the protocol described by Freshney [43]. Trypsin-EDTA solution (0.25%, Sigma-Aldrich) was used to dissociate the C8-D1A cells. Cells from passage 2-15 were used for the experiments.

### 2.2. Stimulation of Astrocytes with the TLR3 Agonist

Twenty-four-hour cultures of C8-D1A cells were treated with a TLR3 agonist, viral dsRNA substitute—poly(I:C) (InvivoGen, San Diego, CA, USA)—or RIG-I/MDA-5 agonist as a control—poly(I:C)/LyoVec (InvivoGen). At the time of treatment, the culture medium was replaced with fresh medium containing poly(I:C) or poly(I:C)/LyoVec. The 10 *μ*g/ml poly(I:C) and 1 *μ*g/ml poly(I:C)/LyoVec concentrations were determined empirically for further experiments.

### 2.3. Antibodies and siRNAs

Primary antibodies used in the study are listed in Table 1. Secondary antibodies used in the study were goat anti-mouse HRP-conjugated IgG (1 : 5000, Santa Cruz Biotechnology), goat anti-rabbit HRP-conjugated IgG (1 : 5000, Santa Cruz Biotechnology), donkey anti-goat HRP-conjugated IgG (1 : 5000, Santa Cruz Biotechnology), horse anti-mouse HRP-conjugated IgG (1 : 3000, CST), and goat anti-rabbit HRP-conjugated IgG (1 : 3000, CST). siRNAs against TLR3, Syk, Hrs, and STAM were purchased from Santa Cruz Biotechnology together with their negative control, siRNA-A.

### 2.4. Western Blot Analysis

At the indicated times or concentrations, astrocytes were processed for protein assays. Cells were lysed with radioimmunoprecipitation assay (RIPA) buffer (Thermo Fisher Scientific, Waltham, MA, USA) supplemented with 1% protease and phosphatase inhibitor cocktail (Thermo Fisher Scientific), and protein concentration was determined with the Pierce BCA Protein Assay Kit (Thermo Fisher Scientific) and spectrophotometry on an Epoch BioTek spectrophotometer. Proteins (20 *μ*g/well) from the cells were separated by SDS-PAGE and electrotransferred onto PVDF membranes using the Bolt® System (Thermo Fisher Scientific). After blocking for 2 h in phosphate-buffered saline with Tween (PBST) containing 5% nonfat milk, the blots were incubated overnight at 4°C with primary antibodies. Subsequently, membranes were washed 3 times after which they were probed with secondary anti-goat or anti-mouse antibodies conjugated to horseradish peroxidase (HRP) (CST, Boston, MA, USA) for 1 h at room temperature and washed 3 times. The Pierce ECL Western Blotting Substrate (Thermo Fisher Scientific) was used to develop, and autoradiography to visualize the protein bands. The intensity of bands was then analyzed using ImageJ software (NIH, Bethesda, MD, USA) and was normalized to GAPDH.

### 2.5. siRNA Transfection

**Figure 2:**
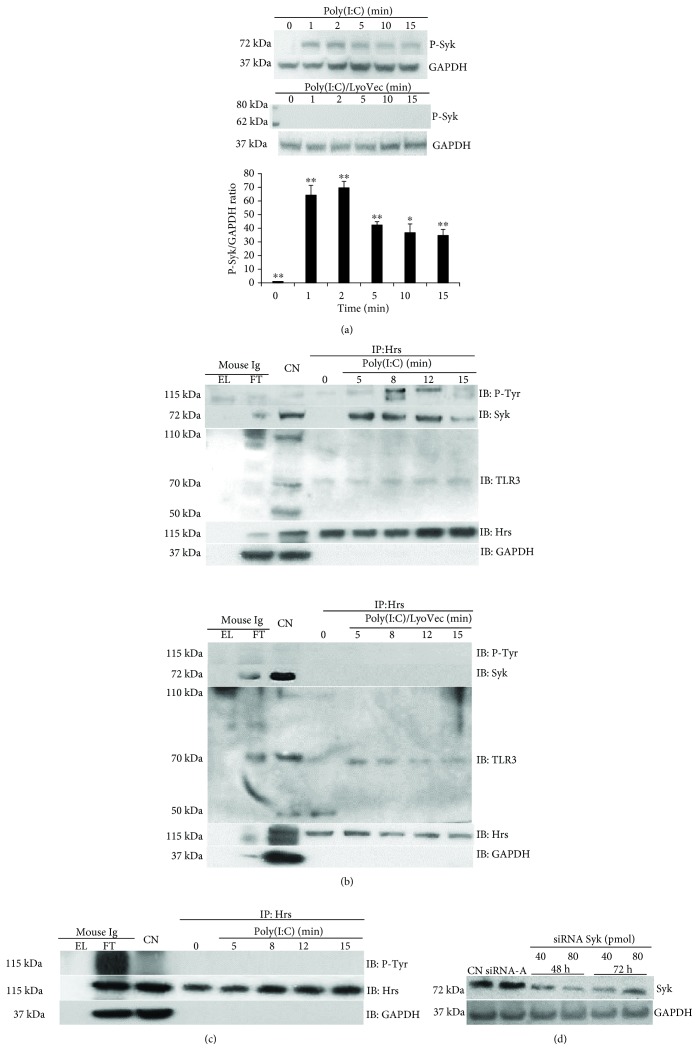
Poly(I:C) treatment of murine astrocytes induces Syk and Hrs phosphorylation and Syk-Hrs interaction. Hrs interacts with the N-terminal cleaved form of TLR3. (a) After poly(I:C) or poly(I:C)/LyoVec stimulation for 1, 2, 5, 10, and 15 min, the phosphorylation of Syk was analyzed by Western blot. The density level of phosphorylated Syk was normalized to GAPDH. Data was obtained from three independent experiments and presented as mean ± SD. ^∗^*p* ≤ 0.05 and ^∗∗^*p* ≤ 0.01. (b) After poly(I:C) or poly(I:C)/LyoVec stimulation for 5, 8, 12, and 15 min, C8-D1A cells were lysed and Hrs was immunoprecipitated using the anti-Hrs antibody. Phosphotyrosine (P-Tyr), Syk, and TLR3 were then detected by Western blot. (c) Following transfection with Syk siRNA, cells were stimulated with poly(I:C) for 5, 8, 12, and 15 min and lysed and Hrs was immunoprecipitated using the anti-Hrs antibody. Phosphotyrosine (P-Tyr) was detected by Western blot. For all immunoprecipitation experiments, 0 min presents untreated cells and mouse IgG were used as a negative control. EL: immunoprecipitation eluate; FT: immunoprecipitation flow-through; CN: control cell lysate. GAPDH was used as protein loading control. (d) Syk silencing efficiency was visualized by immunoblotting with anti-Syk antibodies.

**Figure 3:**
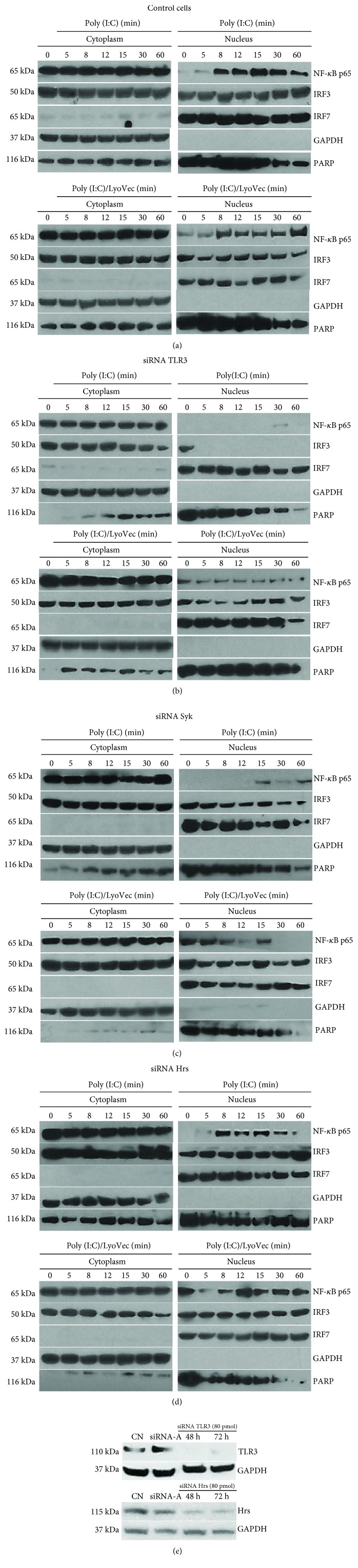
NF-*κ*B nuclear translocation is downregulated in poly(I:C)-treated astrocytes with silenced Syk and Hrs. C8-D1A cells were untreated or treated with poly(I:C) or poly(I:C)/LyoVec for 5 min, 8 min, 12 min, 15 min, 30 min, and 60 min. In advance to the stimulation, astrocytes were not transfected (a) or transfected with siRNA pools for TLR3 (b), Syk (c), and Hrs (d). Following poly(I:C) or poly(I:C)/LyoVec treatment, cytoplasmic and nuclear extracts were immunoblotted with anti-NF-*κ*B p65, -IRF3, -IRF7, -GAPDH, and -PARP antibodies. (e) TLR3 and Hrs silencing efficiency was visualized by immunoblotting with anti-TLR3 and -Hrs antibodies.

**Figure 4:**
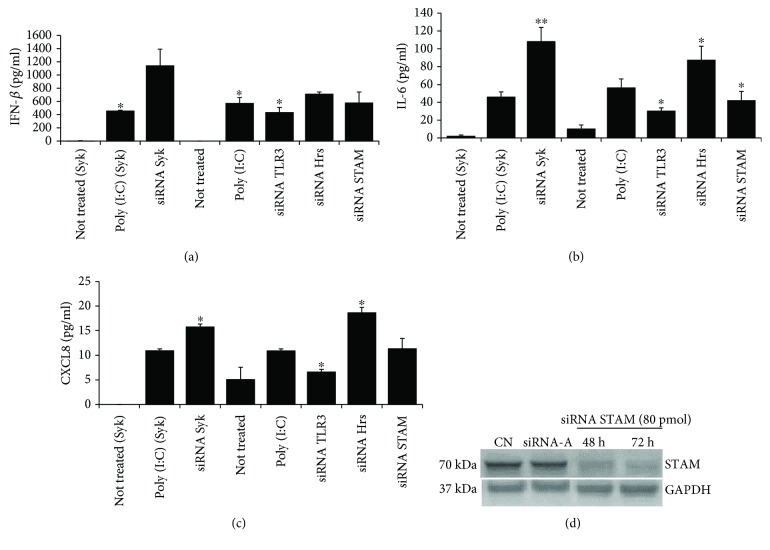
Knockdown of Syk and Hrs expression by siRNA upregulates poly(I:C)-induced IFN-*β*, IL-6, and CXCL-8 production. C8-D1A cells were transfected with control siRNA-A or siRNA pools for TLR3, Syk, Hrs, and STAM. Following the transfection, astrocytes were treated with poly(I:C) (10 *μ*g/ml) for 24 h. IFN-*β* (a), IL-6 (b), and CXCL-8 (c) were measured in culture supernatants by ELISA. Because Syk transfection lasted 48 h, in each experiment, supernatants from untreated (not treated (Syk)), poly(I:C)-treated (poly(I:C) (Syk)), and poly(I:C)-treated cells with silenced Syk (siRNA Syk) were tested in the group independent from cells with silenced TLR3, Hrs, and STAM, where transfection lasted 72 h. (d) STAM silencing efficiency was visualized by immunoblotting with anti-STAM antibodies. Data was obtained from three (IFN-*β*, CXCL-8) or five (IL-6) independent experiments and presented as mean ± SD. ^∗^*p* ≤ 0.05 and ^∗∗^*p* ≤ 0.01.

Twenty-four hours prior to transfection with TLR3, Syk, Hrs, and STAM siRNA, 2 × 10^5^astrocytes per well were seeded in a 6-well plate in the antibiotic-free normal growth medium. For each transfection, 40 or 80 pmol of the specific siRNA was added to 8 *μ*l of the Transfection Reagent (Santa Cruz Biotechnology) to obtain Transfection Reagent mixture according to the manufacturer's instructions. Following 45 min incubation of the mixture at room temperature, cells were washed with Transfection Medium (Santa Cruz Biotechnology) and then the Transfection Reagent mixture was overlaid onto the washed cells. After 7 h incubation, a normal growth medium containing 2 times the normal serum was added to the cells without removing the transfection mixture. Astrocytes were cultured for 48 h or 72 h from the beginning of the transfection, and the efficiency of each of the siRNA transfection was affirmed by western blotting (see Figures 2(d), 3(e), and 4(d)).

### 2.6. Immunostaining and Fluorescence Microscopy

Astrocytes were cultured on 22 mm glass cell culture coverslips in 24-well plates. Untreated or poly(I:C)-treated cells were fixed for 15 min in phosphate-buffered saline (PBS) with 4% paraformaldehyde (Sigma-Aldrich). After washing, cells were permeabilized with 0.5% Triton X-100 (Sigma-Aldrich) in PBS and blocked with 3% bovine serum albumin (Sigma-Aldrich) with 0.1% Triton X-100. Subsequently, cells were incubated for 1 h with anti-TLR3 or anti-STAM or anti-Syk or anti-Hrs antibodies (1 : 50, Thermo Fisher Scientific) and washed with 0.1% Triton X-100 in PBS. Then, astrocytes were incubated with secondary antibodies conjugated with rhodamine Red-X (1 : 100, Jackson ImmunoResearch Laboratories Inc., West Grove, PA, USA) for 1 h. In double immunofluorescence experiments, cells were incubated for 1 h with the mixture of anti-TLR3 and anti-PDI antibodies or anti-STAM and anti-PDI antibodies. Next, after washing, cells were incubated for 1 h with a mixture of secondary antibodies conjugated with rhodamine Red-X or FITC (Jackson ImmunoResearch Laboratories). The cells were then washed and stained with Hoechst 33342 (Sigma-Aldrich) for 10 min. Finally, after washing with PBS, the coverslips were mounted in ProLong Gold Antifade Reagent (Thermo Fisher Scientific). Fluorescence microscopy was performed with an Olympus BX60 fluorescence microscope and analyzed with Cell^F software (Soft Imaging System) (Olympus, Tokyo, Japan).

### 2.7. Immunoprecipitation

C8-D1A cells were cultured in a 6-well plate to reach 80-100% confluence. Cells were stimulated with poly(I:C) or poly(I:C)/LyoVec at the indicated times. If the transfection with specific siRNA was required, astrocytes were pretransfected and 48 or 72 h posttransfection stimulated with poly(I:C) or poly(I:C)/LyoVec as described in Section 2.2. Subsequently, the cells were lysed with IP lysis buffer (Thermo Fisher Scientific) supplemented with protease and phosphatase inhibitor cocktail (Thermo Fisher Scientific). Syk, Hrs, STAM, and TLR3 were immunoprecipitated using Catch and Release® v2.0 Reversible Immunoprecipitation System (Merck) according to the manufacturer's protocol and subjected for immunoblotting. Normal mouse IgG (Santa Cruz Biotechnology) was used as negative control for immunoprecipitation experiments.

### 2.8. Enzyme-Linked Immunosorbent Assay (ELISA)

For the evaluation of IFN*β*, IL-6, and CXCL8 secretion, C8-D1A cells were seeded in a 6-well plate and transfected with specific siRNA (for TLR3, Hrs, STAM, or Syk) or siRNA-A as a negative control. The subsequent concentration of siRNA and duration of the transfection were based on the result, which corresponded to the best knockdown efficiency of the specific protein (see Section 2.4). Following the transfection, cells were detached and seeded in a 24-well plate at a density of 3 × 10^5^ cells per well in a 0.5 ml normal growth medium and treated with polyI:C or poly(I:C)/LyoVec or not treated. After 24 h, the cell supernatants were harvested after centrifugation at 1000 x*g* for 5 min and stored at -80°C until analysis in ELISA assays. The tested proteins were measured with mouse IFN-beta ELISA Kit (R&D Systems, Minneapolis, MN, USA), mouse IL-6 ELISA Kit (Thermo Fisher Scientific), and mouse CXCL8 ELISA Kit (MyBioSource, San Diego, CA, USA), following the manufacturer's instructions. The measurements of optical densities (OD) were done in a microplate reader (Epoch spectrophotometer, BioTek Instruments Inc., Winooski, VT, USA). Quantification of each cytokine concentration in cell supernatants was determined by reading ODs on a linear calibration curve generated for each protein.

### 2.9. Cell Fractionation

C8-D1A cells were untreated or treated with poly(I:C) or poly(I:C)/LyoVec for 5, 8, 12, 15, 30, and 60 min. Subsequently, cells were collected and fractionated using NE-PER Nuclear and Cytoplasmic Extraction Reagents (Thermo Fisher Scientific) according to the manufacturer's protocol. Nuclear and cytoplasmic extracts' protein concentration was determined using BCA Protein Assay kit (Thermo Fisher Scientific). Next, nuclear and cytoplasmic extracts were subjected to western blot analysis.

### 2.10. Statistical Analysis

Quantitative data are presented as mean ± standard deviation (SD) from at least three independent biological experiments (unless otherwise indicated). All data were analyzed in STATISTICA software (StatSoft, Poland). Comparisons were made using Student's *t*-test. A *p* value ≤0.05 (^∗^) or ≤0.01 (^∗∗^) was considered statistically significant.

## 3. Results

### 3.1. TLR3 Undergoes Cleavage upon Poly(I:C) Stimulation of Murine Astrocytes

**Figure 5:**
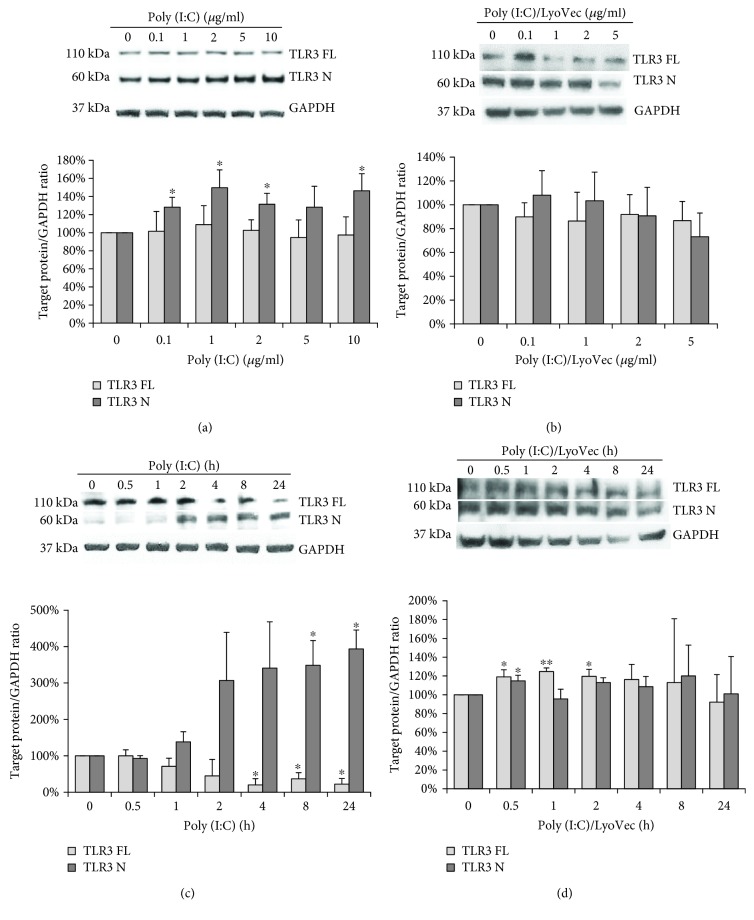
TLR3 of murine astrocytes is cleaved upon stimulation of cells with poly(I:C). Representative western blots of TLR3 expression in C8D1A cells treated with various concentrations of poly(I:C) (0, 0.1, 1, 2, 5, and 10 *μ*g/ml) (a) or poly(I:C)-LyoVec (0, 0.1, 1, 2, and 5 *μ*g/ml) (b) and lysed 24 h after stimulation. TLR3 expression was also analyzed in cells treated with poly(I:C) at concentration 10 *μ*g/ml (c), or with poly(I:C)-LyoVecat concentration 1 *μ*g/ml (d), and lysed at various times of stimulation (0, 30 min, 1 h, 2 h, 4 h, 8 h, and 24 h). TLR3 FL: full-length TLR3; TLR3 N: cleaved N-terminal TLR3 form; GAPDH: protein loading control. Densitometry analysis of TLR3 forms was performed in cells treated with indicated poly(I:C) concentrations for 24 h (a), indicated poly(I:C)/LyoVecconcentrations for 24 h (b), 10 *μ*g/ml poly(I:C) for indicated time points (c), or 1 *μ*g/ml poly(I:C)/LyoVec for indicated time points (d). The density level of each protein was normalized to GAPDH. Data was obtained from three independent experiments and presented as mean ± SD. ^∗^*p* ≤ 0.05 and ^∗∗^*p* ≤ 0.01.

TLR3 belongs to the subfamily of TLRs that reside in endosomes. Maintaining appropriate conditions in the interior of these structures not only serves for appropriate ligand recognition by TLR3 [44] but also enables cleavage by pH-dependent cysteine proteases—cathepsins B, H, L, and/or S [45, 46]. To evaluate whether astrocytic TLR3 responds to poly(I:C) treatment and undergoes proteolytic processing, we treated cells with poly(I:C) at different concentrations or with poly(I:C)/LyoVec (10 *μ*g/ml) for various time intervals and performed western blot analysis with the antibodies directed against amino acid fragment localized at the N-terminus of the TLR3 protein. Figures 5(a)–5(d) show that in C8-D1A cells, the receptor occurs in full-length (TLR3 FL) and N-terminal (TLR3 N) progeny form. Demonstration of the TLR3 N was possible due to the use of the monoclonal TLR3 antibody recognizing amino acids 55-70 of the TLR3 protein. Further, the cleavage of TLR3 is dose-dependent (Figure 5(a)) and time-dependent (Figure 5(c)) upon stimulation with poly(I:C). A similar outcome was not observed in the case of astrocytes treated with poly(I:C)/LyoVec, where the dose (Figure 5(b)) and the time of stimulation (Figure 5(d)) did not affect the expression levels of both FL and N forms of TLR3. Western blot data largely agreed with the densitometric data showing statistically significant (*p* ≤ 0.05) differences in amounts of cleaved TLR3 depending on dose (Figure 5(a)) and time (Figure 5(c)) of exposure of cells to the TLR3 agonist.

### 3.2. Expression of Syk and Hrs Is Upregulated upon Stimulation of Murine Astrocytes with Poly(I:C)

**Figure 6:**
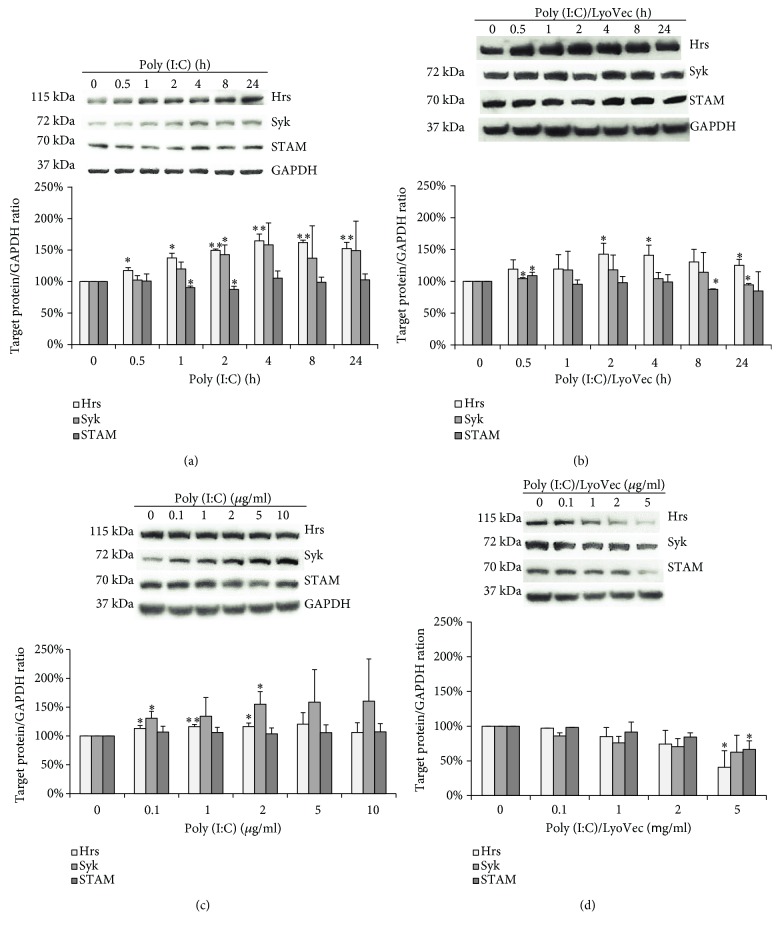
Stimulation of murine astrocytes with poly(I:C) leads to the time-dependent increase in Syk and Hrs expression, while the expression of STAM does not significantly change after stimulation of cells with the TLR3 ligand. Representative western blots of Hrs, Syk, and STAM expression in C8D1A cells treated with various concentrations of poly(I:C) (0, 0.1, 1, 2, 5, and 10 *μ*g/ml) (a), or poly(I:C)-LyoVec (0, 0.1, 1, 2, and 5 *μ*g/ml) (b), and lysed 24 h after stimulation. Hrs, Syk, and STAM expression was also analyzed in cells treated with poly(I:C) at concentration 10 *μ*g/ml (c), or with poly(I:C)-LyoVecat concentration 1 *μ*g/ml (d), and lysed at various times of stimulation (0, 30 min, 1 h, 2 h, 4 h, 8 h, and 24 h). GAPDH was used for evaluating protein loading control. Densitometry analysis of Hrs, Syk, and STAM was performed in cells treated with indicated poly(I:C) concentrations for 24 h (a), indicated poly(I:C)/LyoVecconcentrations for 24 h (b), 10 *μ*g/ml poly(I:C) for indicated time points (c), or 1 *μ*g/ml poly(I:C)/LyoVec for indicated time points (d). The density level of each protein was normalized to GAPDH. Data was obtained from three independent experiments and presented as mean ± SD. ^∗^*p* ≤ 0.05 and ^∗∗^*p* ≤ 0.01.

Recent studies have reported that Syk may be an important controller of the immune receptor transportation and signaling [38, 47, 48] and that Hrs participates in the regulation of responses of multiple TLRs [27, 49]. Importantly, Hrs may contribute to protein cargo sorting as the HRS-STAM heteromer, homotypic hexamer [50], or in combination with subunits from other ESCRT complexes, e.g., with tumor susceptibility gene 101 (TSG101), the ESCRT-I component [51]. Therefore, we investigated if stimulation of astrocytes with the TLR3 ligand entails changes in the level of Syk, Hrs, and STAM expression, which could point to the potential role of these proteins in TLR3 immune modulation. Following TLR3 stimulation, Syk and Hrs expression was upregulated in a time-dependent manner (Figure 6(a)), and densitometric measurements showed highly statistically significant (*p* ≤ 0.01) difference for Hrs. Similarly, following poly(I:C)/LyoVec treatment, the Hrs expression level also increased, but not to such a high level as following poly(I:C) treatment (Figure 6(b)). On the contrary, expression of Syk and STAM remained at a similar level throughout all stimulation time intervals with poly(I:C)/LyoVec (Figure 6(b)). We also observed a significant increase in the expression of Syk in response to treatment with increasing concentrations of poly(I:C), in contrast to Hrs (Figure 6(c)). All the studied proteins exhibited reduced expression level when astrocytes were treated with poly(I:C)/LyoVec at concentrations of 2 and 5 *μ*g/ml, which could indicate that these proteins may not participate in MDA5 signaling in murine C8-D1A cells (Figure 6(d)). Interestingly, no significant differences in STAM expression were observed, either after poly(I:C) treatment of astrocytes with various concentrations or at different time courses (Figures 6(a) and 6(c)). Similar results were observed in STAM expression, after stimulation of cells with poly(I:C)/LyoVec (Figures 6(b) and 6(d)).

### 3.3. Distribution of TLR3 and STAM Is Altered upon Poly(I:C) Stimulation of Murine Astrocytes

**Figure 7:**
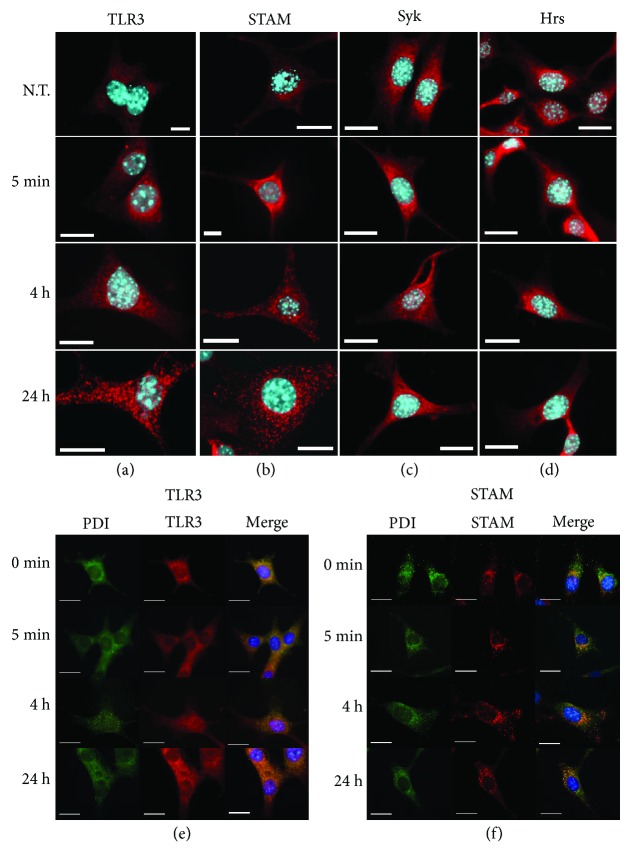
Immunostains of TLR3, Syk, Hrs, and STAM expression and localization in C8D1A cells after treatment with poly(I:C). C8-D1A murine astrocytes were not treated or treated with poly(I:C) (10 *μ*g/ml) for 5 min, 4 h, and 24 h, fixed and immunostained with specific antibodies. Selected images present intracellular distribution of TLR3 (a), Syk (b), Hrs (c), and STAM (d) (red fluorescence). To visualize colocalization of ER with TLR3 or STAM, following poly(I:C) stimulation at the indicated time points, cells were double stained with anti-TLR3 (red) and anti-PDI (green) antibodies (e), or with anti-STAM (red) and anti-PDI (green) antibodies (f). Nuclear DNA was stained with Hoechst 33342 (blue fluorescence). Scale bar = 10 *μ*m, N.T. = no treatment.

To study whether the observed changes have a reflection in protein distribution at the cellular level following stimulation and thus better understand the connection between TLR3, ESCRT-0, and Syk in astrocytes, we examined the localization of TLR3, Syk, Hrs, and STAM in C8-D1A cells stimulated with poly(I:C) (10 *μ*g/ml) for 5 min, 4 h, and 24 h. We chose such a time scale to show that the posttranslational modifications of Syk and Hrs that occur within the first half hour after TLR3 stimulation are not significantly related to the change in their distribution. Consequently, noticeable modification in the arrangement of TLR3 and STAM was found at 4 h and 24 h after the addition of poly(I:C) (Figures 7(a) and 7(b)). Our findings indicate that TLR3, STAM, Syk, and Hrs are highly expressed in murine astrocytes (Figures 7(a)–7(d)). To validate the colocalization of TLR3 and STAM with ER, we performed staining of the ER with the anti-protein disulfide isomerase (anti-PDI) antibody (Figures 7(e) and 7(f)). TLR3 and STAM exhibited distinct distribution patterns in indicated time courses of poly(I:C) stimulation. In nontreated cells (resting cells), TLR3 was localized near the cell nucleus, most likely in the ER, whereas after stimulation it gradually dispersed until it was evenly distributed throughout the cell following 24 h of poly(I:C) treatment (Figures 7(a) and 7(e)). Similarly, the majority of STAM was present in the perinuclear region in untreated cells, whereas in poly(I:C)-stimulated cells, the longer the duration of stimulation, the larger the dispersion and abundance of the protein vesicles was observed (Figures 7(b) and 7(f)). We observed a similar distribution of Syk and Hrs in untreated cells and cells stimulated with poly(I:C) (Figures 7(c) and 7(d)); most of the proteins were located near the nucleus, while part was dispersed in the cytoplasm.

### 3.4. Stimulation of Cells with the TLR3 Ligand Promotes Syk Activation and Leads to Syk-Hrs Interaction, Tyrosine Phosphorylation of Hrs, and Hrs Interaction with the N-Terminal Cleaved Form of TLR3

Knowledge regarding factors that contribute to the activation of Syk in TLR signaling still needs to be broadened; however, it has been indicated that TLR ligands are capable of inducing Syk activation [52]. Furthermore, it has been demonstrated that Hrs is the target of Syk activity during high-affinity IgE receptor (Fc*ε*RI) endocytosis and that Syk orchestrates Hrs intracellular localization—cytosolic Hrs is ubiquitinated, while Hrs phosphorylation leads to the transfer of Hrs to the membrane compartments [39]. Here, we demonstrate that poly(I:C) stimulation of astrocytes leads to rapid phosphorylation of Syk, which appears at 1 min and peaks at 2-5 min following TLR3 stimulation (Figure 2(a)). Western blot data corresponded with the densitometric analysis where the highest level of Syk phosphorylation was observed 1-2 min after the addition of poly(I:C) (Figure 2(a)). Such a result is consistent with the work of Cao et al. [53], where phosphorylation of the Tyr-346 residue of Syk was detected at 1 min, and a maximal Syk phosphorylation increase was observed at 5 min poststimulation of Fc*ε*RI in MCP5 cells. We have not observed Syk phosphorylation after the addition of poly(I:C)/LyoVec to the cells (Figure 2(a)). To investigate whether Syk could associate with Hrs, lysates from astrocytes stimulated or unstimulated with poly(I:C) for the indicated time points were subjected to immunoprecipitation with anti-Hrs antibody and probed with anti-Syk antibody. In the fifth minute after the addition of the TLR3 ligand, we detected an interaction of Syk and Hrs (Figure 2(b)), which peaked between 5 and 12 min and decreased within 15 min of stimulation, whereas such interaction did not occur after the addition poly(I:C)/LyoVec to the cells (Figure 2(b)). A similar result was observed after Fc*ε*RI activation, where the Syk-Hrs interaction was maximal at 5 min and decreased to near-baselinewithin 20 min of stimulation [39]. Next, we evaluated whether Hrs may serve as a substrate for Syk-mediated phosphorylation. Lysates from unstimulated or poly(I:C)-stimulated astrocytes were subjected to immunoprecipitation with anti-Hrs antibody and probed with anti-phosphotyrosine antibody. Stimulation of cells with poly(I:C) resulted in tyrosine phosphorylation of Hrs (Figure 2(b)). Importantly, Hrs phosphorylation peaked at 5-12 min following the addition of poly(I:C), which is consistent with the peak of the Syk-Hrs interaction. Hrs phosphorylation was undetectable in cells stimulated with poly(I:C)/LyoVec (Figure 2(b)). We did not observe phosphorylation of Hrs after the addition of poly(I:C) to the cells treated with siRNA against Syk (Figure 2(c)), indicating that Syk may be the crucial kinase responsible for Hrs modification. Furthermore, following immunoprecipitation of astrocytic lysates with anti-Hrs antibody and immunoblotting with antibody recognizing amino acids at the N-terminal part of TLR3, we observed that the TLR3 N-terminal progeny form interacts in increasing amounts with Hrs following poly(I:C) stimulation (Figure 2(b)).

### 3.5. Poly(I:C) Treatment of Murine Astrocytes Induces TLR3 Tyrosine Phosphorylation and Promotes Interaction with Hrs

**Figure 8:**
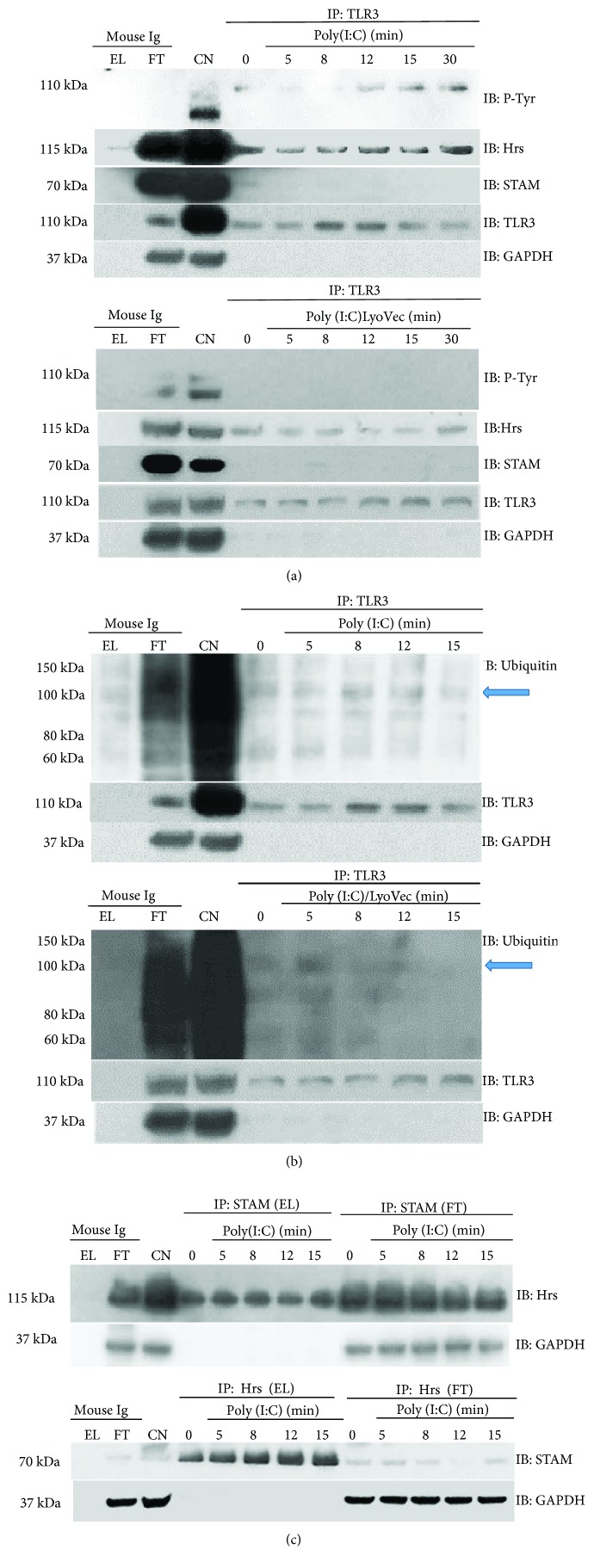
Poly(I:C) treatment of murine astrocytes induces TLR3 tyrosine phosphorylation and promotes interaction with Hrs. (a) After poly(I:C) or poly(I:C)/LyoVec stimulation for 5, 8, 12, 15, and 30 min, C8-D1A cells were lysed and TLR3 was immunoprecipitated using the anti-TLR3 antibody. Phosphotyrosine (P-Tyr), Hrs, and STAM were then detected by Western blot. (b) Following poly(I:C) or poly(I:C)/LyoVec stimulation for 5, 8, 12, andn 15 min, C8-D1A cells were lysed and TLR3 was immunoprecipitated using the anti-TLR3 antibody. Ubiquitin was detected by Western blot. Blue arrows indicate ubiquitinated TLR3. (c) Following poly(I:C) stimulation for 5, 8, 12, and 15 min, murine astrocytes were lysed and STAM and Hrs were immunoprecipitated using anti-STAM and anti-Hrs antibodies, respectively. Hrs and STAM were detected by Western blot. For all immunoprecipitation experiments, 0 min presents untreated cells and mouse IgG were used as a negative control. EL: immunoprecipitation eluate; FT: immunoprecipitation flow through; CN: control cell lysate. GAPDH was used as protein loading control.

Previous studies have identified Hrs as the protein that directly interacts with TLRs such as TLR2, TLR4, TLR7, and TLR9 [27, 32, 49]. To investigate whether Hrs associates with TLR3, lysates from astrocytes unstimulated or stimulated with poly(I:C) were subjected to immunoprecipitation with anti-TLR3 antibody. Probing of the immunoblot with anti-Hrs antibody showed that Hrs bound to TLR3 in nonstimulated cells. However, an increasing proportion of Hrs associated with TLR3 in a time-dependent manner (Figure 8(a)) in poly(I:C) stimulated cells. A similar result was observed in the case of TLR4, the receptor constitutively associated with Hrs, and the interaction increased after TLR4 stimulation [32]. Following poly(I:C)/LyoVec cell stimulation, only a small amount of Hrs was associated with TLR3 (Figure 8(a)). Similarly, following TLR3 immunoprecipitation, we probed immunoblots with anti-STAM antibody and determined whether the second ESCRT-0 subunit interacts with TLR3. Interestingly, we did not observe any interaction between TLR3 and STAM, either following poly(I:C) or poly(I:C)/LyoVec stimulation of astrocytes (Figure 8(a)). Furthermore, we wanted to address if poly(I:C) induces TLR3 ubiquitination. By probing western blots of immunoprecipitated TLR3 with anti-ubiquitin antibody, we showed that TLR3 is similarly ubiquitinated in C8-D1A cells treated with poly(I:C) or poly(I:C)/LyoVec (Figure 8(b)). Such modification of TLR3 may condition the interaction with ubiquitin-binding molecules, e.g., Hrs. Because ubiquitin ligases are known to interact with tyrosine-phosphorylated proteins, we examined the level of TLR3 phosphorylation in nonstimulated and poly(I:C)-stimulated cells. Phosphorylation of the receptor was observed 8 min after the addition of poly(I:C) and later (Figure 8(a)), which may indicate that TLR3 modification such as ubiquitination is not fully dependent on the receptor's phosphorylation. Finally, we investigated whether Hrs-STAM interaction may be promoted by stimulation of cells with poly(I:C). While the majority of the Hrs cellular pool remained unassociated with STAM, the greater part of STAM interacted with Hrs in a time-dependent manner following TLR3 stimulation (Figure 8(c)). Such a result indicates that Hrs may not reside in the same cellular localization as STAM; however, the addition of poly(I:C) may foster interaction between these proteins.

### 3.6. Changes in the Intracellular Localization of NF-*κ*B, IRF3, and IRF7 in TLR3-, Syk-, and Hrs-Depleted Astrocytes following TLR3 Stimulation

After describing molecular interactions between Syk and Hrs, as well as TLR3, Syk, and Hrs posttranslational modifications following poly(I:C) stimulation of C8-D1A cells, we investigated the role of Syk and Hrs in poly(I:C)-induced NF-*κ*B, IRF3, and IRF7 nuclear translocation. Following the addition of poly(I:C) to the control cells, we observed translocation of NF-*κ*B and IRF3 to the nucleus (Figure 3(a)). In contrast, NF-*κ*B and IRF3 did not accumulate in the nucleus of TLR3-depleted cells stimulated with poly(I:C) (Figure 3(b)). Furthermore, NF-*κ*B nuclear translocation was downregulated in poly(I:C)-treated astrocytes with knocked-down Syk, and shortage of Syk also appeared to minimally affect nuclear translocation of IRF3 in C8-D1A cells (Figure 3(c)). In Hrs-depleted cells NF-*κ*B activation was reduced to a certain extent after stimulation with poly(I:C), whereas translocation of IRF3 to the nucleus remained intact in comparison to the control cells (Figure 3(d)). Interestingly, the amount of IRF7 localized in the nucleus was similar throughout the duration of poly(I:C) or poly(I:C)/LyoVec stimulation, and its physiological level was also observed in the nontreated cells (0 min time point, Figures 3(a)–3(d)).

### 3.7. A Role for Syk, Hrs, and STAM in the Regulation of TLR3 Signaling

To verify the influence of Syk, Hrs, and STAM knockdown on the response of C8-D1A cells to TLR3 ligand stimulation, we transfected astrocytes with the specific siRNAs and subsequently activated the cells with poly(I:C). Following cell stimulation, secreted IFN*β*, IL-6, and CXCL8 were quantified by ELISA. In Syk-knockdown cells, poly(I:C) significantly increased secretion of IFN*β* (*p* ≤ 0.05), IL-6 (*p* ≤ 0.01), and CXCL8 (*p* ≤ 0.05) compared to poly(I:C)-stimulated cells without Syk depletion (Figures 4(a)–4(c)). Knockdown of Hrs in C8-D1A cells significantly enhanced poly(I:C)-dependent induction of IFN-*β*, IL-6, and CXCL8 secretion (*p* ≤ 0.05), while levels of IFN*β* and CXCL8 in STAM-depleted cells stimulated with poly(I:C) were not significantly different from those in poly(I:C)-treated controls, apart from IL-6 (*p* ≤ 0.05) (Figures 4(a)–4(c)). Collectively, these results indicate that Syk and Hrs are involved in TLR3-mediated signaling events. Because Syk and Hrs knockdown results in the increase of IFN-*β*, IL-6, and CXCL8 secretion, Syk and Hrs may serve to regulate TLR3-mediated immune response in intact astrocytes.

## 4. Discussion

In our study, we reveal that astrocytic TLR3 undergoes proteolytic processing and that the expression of TLR3 N increases in proportion to the poly(I:C) dose and length of exposure. Presentation of TLR3 in such a manner may modulate the level of response to viral dsRNA, especially in CNS cells. For example, such TLR3 form may constitute a negative regulator of signaling, as found for TLR9 [54], but this requires further investigation. TLR3 present in mammalian cells has a size of approximately 110 kDa; however, cleaved TLR3 molecules have been shown to remain associated and are also capable of binding the TLR3 ligand [55, 56]. Such TLR3 configuration may represent the primary form of the receptor, and it is possible that the cleavage is aimed at actuating the novel receptor attributes, other than separation of the two progeny fragments. Conclusions from previous studies on the role of TLR3 cleavage are ambiguous. Proteolytic processing of TLR7 or 9 by cathepsins is required for signaling; however, TLR3 cleavage may not determine the activation of the immune response [57]. The addition of the cathepsin inhibitor or mutation at the TLR3 cleavage site did not influence TLR3 response to poly(I:C) in 4 cell lines, although inhibition of cleavage decreased the abundance of the receptor to be degraded in lysosomes [46]. On the other hand, TLR3 cleavage was indispensable for the receptor activation in murine RAW cells [58]. Zhang et al. [59] linked mutation situated in the region of the TLR3 cleavage and critical for dsRNA binding (P554S) in a patient suffering from HSE with the loss of TLR3 function in CNS cells and increased penetrance of the disease through insufficient antiviral response. This highlights the importance of proper TLR3 cleavage and its possible influence on ligand recognition and activation of the signaling pathway.

Hrs and STAM are components of the ESCRT-0 transportation complex; however, they also function as separate proteins, e.g., Hrs bound in the membrane occurs partly as a monomer and partly as a form associated with STAM [50]. Hrs appears to be only partially involved in cooperation with STAM in C8-D1A cells not stimulated or stimulated with poly(I:C) (Figure 8(c)), and we did not observe any significant differences in the TLR3-mediated immune response level following STAM depletion (Figures 4(a)–4(c)). This indicates that after TLR3 activation in astrocytes, Hrs may interact with proteins from complexes other than ESCRT-0. ESCRT-0 subunits, alone or in combination with other proteins, may moonlight in manifold activities; e.g., Hrs in cooperation with the product of tumor susceptibility gene 101 (*TSG101*), an ESCRT-I subunit, leads to the endocytic downregulation of EGFR [51]. This protein is a tyrosine kinase responsible for the phosphorylation of Tyr858 of TLR3, a modification indispensable for the recruitment of TRIF [36]. Silencing of Hrs, resulting in the reduced degradation of EGFR and concomitantly increased activatory influence on TLR3, could manifest in an increase of TLR3-mediated innate response to poly(I:C) and possibly viral dsRNA. Furthermore, we have demonstrated that TLR3 interacts with Hrs, which may have a direct influence on the fate of the receptor. Hrs may be involved in directing TLR3 N to the degradative pathway, as is the case with various cell membrane receptors. This, particularly, appears likely because IFN*β*, IL-6, and CXCL8 secretion following knockdown of Hrs was higher compared to cells with intact Hrs. In the noncanonical ESCRT-0 pathway, which does not engage MVBs or lysosomes, Hrs has been implicated in targeting of ubiquitinated TLR9 and TLR7 to endosomes for ligand recognition. Furthermore, Hrs silencing blocked the nuclear transportation of NF-*κ*B p65 and reduced the level of TNF*α* and IFN*α* secretion following TLR9 stimulation [27]. We performed a similar experiment to examine NF-*κ*B activation in the astrocytes with the knockdown of Hrs. Following TLR3 stimulation, NF-*κ*B exhibited reduced but not abolished transportation to the cell nucleus. However, we did not observe a significant influence of Hrs knockdown on the nuclear accumulation of IRF-3 or IRF-7, suggesting that Hrs neither is a key moderator nor participates in signaling steps leading to the intracellular transfer of these transcription factors. Furthermore, knockdown of Syk and Hrs in C8-D1A cells increased TLR3-dependent IFN*β*, IL-6, and CXCL8 secretion following stimulation with poly(I:C). This suggests that Syk and Hrs participate in TLR3-mediated innate response to dsRNA; however, it is likely that Hrs potentially engages in the signaling events not related to TLR3 endosomal trafficking aimed at ligand detection, as in the case of TLR7 and TLR9. Syk may play a dual role in TLR4 activation by promoting the signaling through mediation of endocytosis or inhibiting signaling from the plasma membrane [60], similar to Hrs, which supports contradictory cellular events leading to degradation or recycling of numerous receptors. Following LPS sensing, ubiquitinated TLR4 interacts with Hrs on the way to degradation in lysosomes and it is possible that other cell surface TLRs are trafficked in the analogous manner [32]. It should be noted that although we were unable to acquire complete silencing of Syk, the knockdown achieved was adequate to affect Hrs phosphorylation, NF-*κ*B nuclear translocation, or IFN*β*, IL-6, and CXCL8 secretion. We speculate that deprivation of the cells of the vast majority of Syk is sufficient to prevent Hrs phosphorylation, which may indicate Syk as the key protein responsible for Hrs activation. Therefore, the shortage in Syk followed by lack of Hrs phosphorylation may be reflected in subsequent cellular events associated with TLR3 signaling.

Posttranslational modifications play important roles in the regulation of Hrs activity, and Hrs phosphorylation has been proven to correlate with EGFR degradation [33]. Interestingly, astrocytes exhibited phosphorylation of a small proportion of the cellular pool of Hrs (Figure 2(b)), which is consistent with the Hrs phosphorylation level observed downstream of EGFR activation by Stern et al. [33]. If Hrs was implicated in retaining of degradative transportation of TLR3, the depletion of Syk and Hrs would open the possibility of prolonged TLR3 recruitment in the signaling cycle. It is highly probable that following viral dsRNA-mediated stimulation of astrocytes, concurrent with endosomal trafficking, ligand recognition, and execution of the TLR3 signaling cascade, a process leading to ligand-induced degradation of the receptor is initiated with the aim of modulating and maintaining the proinflammatory response at an adequate level.

Indirect immunofluorescence analysis revealed that TLR3 and STAM exhibited distinct staining patterns at different time courses after poly(I:C) stimulation (Figures 7(a) and 7(b)). STAM was found to colocalize with ER, Golgi, and endosomal markers and participate in the reconstruction and restoration of the Golgi structure [28]. Owing to the VHS domain, STAM may interact with the Golgi-localizing proteins and participate in protein sorting at the *trans*-Golgi network [61, 62]. Thus, the distribution pattern of STAM observed after poly(I:C) stimulation may reflect its functions related to intracellular TLR3 trafficking; however, this requires further investigation. Another unique attribute of STAM is the diphosphorylated immune tyrosine activation motif (ITAM) [63], the distinctive sequence found in the cytoplasmic subunits of B and T cell receptors, as well as Fc receptors. Upon phosphorylation, ITAM serves as a ligand for SH2 domains of various cytoplasmic tyrosine kinases, e.g., Syk. Following docking at ITAM, kinase undergoes conformational changes, resulting in autophosphorylation of the Syk catalytic domain. Such an event increases Syk enzymatic activity and leads to propagation of downstream signaling [64, 65]. We used specific anti-Syk mouse (pY342) antibodies to confirm that conserved tyrosine Y-342 in the Syk SH-2 linker region was phosphorylated following TLR3 activation (Figure 2(a)). Mutation of this particular tyrosine residue alone was found to diminish Syk ability to interact with other proteins, while a concomitant mutation in Y-346 induced a significant reduction in Fc*ε*RI-mediated signaling [66]. Lin et al. [52] confirmed that stimulation with poly(I:C) led to the activation of Syk in bone marrow-derived macrophages (BMDM) and RAW.264.7 macrophages; however, phosphorylation of the kinase was detected 15 min after stimulation of the cells. We have shown that Syk undergoes rapid phosphorylation and associates with Hrs after poly(I:C) stimulation of astrocytes (Figures 2(a) and 2(b)); however, it cannot be precluded that such interaction occurs with the participation of other proteins. Further, studies are necessary to confirm whether STAM may associate with the phosphorylated SH-2 domain of the Syk *via* its ITAM region, although the ITAM-independent pathway may underlay Syk-Hrs interaction.

The possibilities where Syk may affect TLR responses are manifold; in this work, we addressed the ways in which Syk could influence TLR3 signaling and cytokine responses in cells of brain origin. Syk may play reverse roles in mediating TLR-dependent responses by opposingly regulating the ubiquitination of TRAF3 or TRAF6 [60]. Furthermore, Syk might inhibit MyD88-dependent production of proinflammatory cytokines and augment the TRIF-dependent expression of IFN-dependent genes. Despite the fact that Syk-mediated TRIF phosphorylation leads to TRIF proteasomal degradation resulting in downregulation of TLR signaling [38], phosphorylation of this adaptor protein is critical for the activation of the type I IFN pathway [67]. Thus, the observed restriction of NF-*κ*B nuclear localization in the absence of Syk (Figure 3(c)) appears to correspond with insufficient activatory influence on the TRIF phosphorylation [67]. Most recently, preclinical and clinical studies highlight pharmacological inhibitors of Syk as promising drug targets, due to their inhibitory influence on the inflammatory responses [52, 68, 69]. Nevertheless, some studies present contradictory findings—Syk deficient cells exhibit higher proinflammatory response than do wild-type cells [70, 71], and such a relationship has been pointed out for Syk-dependent inhibition of TLR signaling [38]. Our data distinguish Syk as the balancing component in TLR3-mediated immune response, intended to avoid unstrained production of inflammatory factors.

TLR3 expression may be modulated by proinflammatory molecules that are upregulated in various neurodegenerative disorders [72]. Recently, the essential role of astrocytes was highlighted in the course of such neurodegenerative diseases as multiple sclerosis (MS), amyotrophic lateral sclerosis (ALS), AD, Parkinson's disease (PD), and HIV-1 associated dementia (HAD) [73–77].

Importantly, recent reports explore and confirm the possible role of HSV-1 infection in the pathogenesis of the most common form of dementia—AD [78, 79]. Therapies targeting glial cells might benefit the cells affected by neurodegenerative disorders. There is good support for the hypothesis that A*β* secreted by cells may constitute an antimicrobial protein (AMP) and astrocytes may produce it as an essential defensive component of the innate immunity [80]. Furthermore, inflammatory agents that appear during both acute and chronic brain infections may upregulate amyloid precursor protein levels in both astrocytes of murine and human brain [81]. During H3N2 and H1N1 influenza A viruses (IAV) or HSV-1 infections, A*β* may play a protective role against these pathogens and constitute a host response to infection, e.g., reduce virus replication in neurons or prevent viral entry into the cells [82, 83]. On the other hand, HSE, and particularly recurrent or persistent HSV-1 infections in the brain, may be the determining factors that increase the risk of AD development. The extent to which HSV-1 infection may contribute to the deposition of A*β* in the brain was analyzed by Wozniak et al. [9]. In brains of people suffering from AD, HSV-1 nucleic acid was found in 90% of A*β* plaques, while over 70% of viral DNA was associated with the plaques. These data indicate HSV-1's presence in the brain as one of the initiating factors in the formation of A*β* plaques in the brain, as well as an important factor that may lead to the onset of AD. Knowledge regarding TLR3 biology in brain cells is significant since the receptor is crucial in combating HSV-1 infection. Results obtained in this work contribute to the understanding of TLR3 functioning in astrocytes.

A functional TLR3 reveals as an essential component of natural immunity to HSV-1 in the brain, while impaired innate immunity to HSV-1 may increase susceptibility to HSE in children and adults. Here, we point out Syk and Hrs as immune factors which influence TLR3 signaling that may affect inflammatory-mediated encephalitic responses during HSE. Details regarding how Syk and Hrs influence TLR3-mediated antiviral response call attention to novel elements which may require careful examination when analyzing the TLR3 activity in CNS, such as the role of posttranslational modifications of these proteins, or contribution of TLR3 N-terminal form in mounting the effective antiviral defense. Precise regulation of the TLR3 transportation and degradation, most likely related to Syk and Hrs, is essential for maintaining the adequate level of an active receptor and generating an effective immune response.

## 5. Conclusions

Endosomal TLR3 undergoes cleavage upon poly(I:C) stimulation of murine astrocytes in a dose- and time-dependent manner. Stimulation of murine astrocytes with poly(I:C) upregulates the expression of Syk and Hrs in a time-dependent manner and additionally in a dose-dependent manner for Syk, while the expression of STAM is not affected. Distribution of TLR3 and STAM is altered, from a perinuclear location in nonstimulated cells to a much dispersed arrangement upon poly(I:C) stimulation of astrocytes. The increased expression of Syk appears to orchestrate its activation and eventual interaction with Hrs followed by tyrosine phosphorylation of Hrs, which in turn interacts with the N-terminal form of TLR3. Knockdown of TLR3, Syk, or Hrs followed by TLR3 stimulation of astrocytes leads to perturbations in nuclear translocation of NF-*κ*B and IRF3, while IRF7 is not influenced. Moreover, Syk and Hrs knockdown results in the increase of IFN*β*, IL-6, and CXCL8 secretion. These results suggest that Syk and Hrs have a regulatory role in signaling through TLR3 in murine astrocytes.

## Figures and Tables

**Table 1:** Primary antibodies used in the western blot assay.

Antibody	Clone/ID	Isotype	Source	Concentration
TLR3	PA5-23105^a^	Polyclonal rabbit	Thermo Fisher Scientific	2 *μ*g/ml
TLR3	MA5-16184^a^	Monoclonal mouse	Thermo Fisher Scientific	2 *μ*g/ml
TLR3	TLR3.7^b^	Monoclonal mouse	OriGene Technologies GmbH	2 *μ*g/ml
Hrs	15087^a^	Monoclonal rabbit	CST	1 : 1000
Hrs	M-79^a^	Polyclonal rabbit	Santa Cruz Biotechnology	1 : 200
Hrs	C-7^a^	Monoclonal mouse	Santa Cruz Biotechnology	1 : 200
STAM	13053^a^	Polyclonal rabbit	CST	1 : 200
STAM	H-175^a^	Polyclonal rabbit	Santa Cruz Biotechnology	1 : 100
STAM	B-2^a^	Monoclonal mouse	Santa Cruz Biotechnology	1 : 200
Syk	N-19^a^	Polyclonal rabbit	Santa Cruz Biotechnology	1 : 200
Syk	4D10^a^	Monoclonal mouse	Santa Cruz Biotechnology	1 : 200
PDI	RL77^b^	Monoclonal mouse	Thermo Fisher Scientific	1 : 100
Phospho-Syk	I120-722^b^	Monoclonal mouse	Becton Dickinson Biosciences	1 : 500
Phosphotyrosine	4G10^a^	Monoclonal mouse	Merck	1 : 1000
Phosphotyrosine	61-5800^a^	Polyclonal rabbit	Thermo Fisher Scientific	1 : 1000
IRF3	4302^a^	Monoclonal rabbit	CST	1 : 1000
NF-ĸB p65	8242^a^	Monoclonal rabbit	CST	1 : 1000
IRF7	PA1-12810^a^	Polyclonal rabbit	Thermo Fisher Scientific	2 *μ*g/mL
PARP	9532^a^	Monoclonal rabbit	CST	1 : 1000
Ubiquitin	P4D1^a^	Monoclonal mouse	Santa Cruz Biotechnology	1 : 200
Ubiquitin	3933^a^	Polyclonal rabbit	CST	1 : 1000
GAPDH	MA5-15738^a^	Monoclonal mouse	Thermo Fisher Scientific	1 : 1000

^a^Manufacturer's antibody identification. ^b^Clone

## Data Availability

All data used to support the findings of this study are included within the article.
